# Low-dose external beam radiotherapy as a postoperative treatment for patients with diffuse pigmented villonodular synovitis of the knee: 4 recurrences in 23 patients followed for mean 9 years

**DOI:** 10.3109/17453674.2012.678803

**Published:** 2012-06-04

**Authors:** Geumju Park, Young Seok Kim, Jong Hoon Kim, Sang-wook Lee, Si Yeol Song, Eun Kyung Choi, Seong Yoon Yi, Seung Do Ahn

**Affiliations:** ^1^Department of Radiation Oncology, Asan Medical Center, University of Ulsan, College of Medicine, Seoul; ^2^Division of Hematology-Oncology, Department of Internal Medicine, Inje University Ilsan Paik Hospital, Goyang, Republic of Korea

## Abstract

**Background and purpose:**

Pigmented villonodular synovitis (PVNS) is a rare proliferative disorder involving synovial membranes, and patients with PVNS have a variable prognosis. We retrospectively analyzed clinical outcomes after synovectomy plus low-dose external beam radiotherapy for diffuse PVNS of the knee.

**Methods:**

We reviewed the medical records of 23 patients who underwent postoperative radiotherapy between 1998 and 2007. 19 patients had primary disease and 4 had recurrent disease with an average of 2.5 prior surgeries. After synovectomy (17 arthroscopic surgeries; 6 open), all 23 patients received 4-MV or 6-MV external beam radiotherapy with a median dose of 20 (12–34) Gy in 10 fractions.

**Results:**

At a median follow-up of 9 (0.8–12) years, 4 patients had recurrent disease, with a median disease-free interval of 5 years. Of these 4 patients, 3 received salvage synovectomy and regained local control. Univariate analysis showed that age, sex, history of trauma, and total dose of radiation were not predictive of local control. 22 patients reported excellent or good joint function, and 1 who refused salvage synovectomy had poor joint function. None of the patients experienced grade 3 or higher radiation-related toxicity or radiation-induced secondary malignancies.

**Interpretation:**

Postoperative external beam radiotherapy is an effective and acceptable modality to prevent local recurrence and preserve joint function in patients with diffuse PVNS of the knee. Low-dose (20 Gy) radiotherapy appears to be as effective as moderate-dose treatment (around 35 Gy).

Pigmented villonodular synovitis (PVNS) is a rare proliferative and destructive disorder involving the synovium of joint capsules, tendon sheaths, and bursae. The estimated annual incidence of PVNS is 1.8 patients per million individuals, and young and middle-aged adults are the most frequently affected (Myers and Masi 1980). PVNS is usually a monoarticular condition, the predominant site being the knee followed by the hip and ankle (Granowitz et al. 1976). The etiology and pathogenesis of PVNS are unknown, but it may be due to chronic inflammation (Oehler et al. 2000) or a neoplastic process (Choong et al. 1995, Somerhausen and Fletcher 2000).

There are two distinct forms of PVNS, localized and diffuse, based on the extent of synovial involvement (Granowitz et al. 1976, Myers and Masi 1980). The two forms are histologically similar, but diffuse PVNS presents with more pronounced symptoms and is more rapidly destructive with a tendency to invade extra-articular structures such as muscles, tendons, bones, neurovascular structures, and skin (Granowitz et al. 1976, O'Sullivan et al. 1995). Whereas successful local control can be achieved by excision of localized masses (Granowitz et al. 1976, Rao and Vigorita 1984), complete tumor removal in patients with the diffuse form may be more difficult, with recurrence rates after surgery alone ranging from 8% to 56% depending on the extent of surgery (Schwartz et al. 1989, Ogilvie-Harris et al. 1992, Flandry et al. 1994, Zvijac et al. 1999). Postoperative radiotherapy (RT) has been used to achieve better local control in patients with primary or recurrent PVNS (O'Sullivan et al. 1995, Blanco et al. 2001, Chin et a. 2002, Lee et al. 2005, Berger et al. 2007, Horoschak et al. 2009, Heyd et al. 2010).

It is unclear whether treatment outcomes are influenced by the location of the disease or the radiation dose. PVNS of the knee is, however, associated with a higher recurrence rate than PVNS at other joints (Schwartz et al. 1989). Moreover, there have been no studies on radiation dose-response relationships to date. At Asan Medical Center, patients have been given the conventional dose (32–34 Gy) or a lower dose (20 Gy) as postoperative treatment for diffuse PVNS of the knee, depending on the views of treating physicians who favored different pathogenesis theories—neoplasia or chronic inflammation. We retrospectively compared the clinical outcomes of patients who were treated with conventional or low-dose RT.

## Patients and methods

### Patient characteristics

We retrospectively reviewed the medical records of 23 consecutive patients who received external beam RT after synovectomy for diffuse PVNS of the knee at Asan Medical Center between 1998 and 2007 (Table 1). The diagnosis in all patients was confirmed histopathologically. Median age was 37 (10–64) years and 15 patients were female. Pain and swelling of the affected knee were the predominant symptoms, with median duration of symptoms of 1.5 (0.5–10) years. 4 patients received postoperative RT for recurrent disease, with 2–3 prior surgeries. MRI was performed in all patients at the initial diagnosis, and at follow-up in 15 patients.

**Table 1.** Patient and treatment characteristics

A	B	C	D	E	F	G	H	I	J	K	L	M
1	28	M	No	P	A	R0	16	No			9	Good
2	48	M	No	P	A	R0	20	No			141	Good
3	14	M	Yes	P	A	R0	20	No			140	Excellent
4	25	F	No	P	A	R2	20	No			140	Good
5	10	F	Yes	P	A	R2	20	Yes	43	Yes	136	Excellent
6	41	F	No	P	O	R2	20	Yes	62	Yes	130	Excellent
7	31	F	No	P	A	R2	20	No			118	Good
8	45	F	No	P	A	R0	20	No			115	Good
9	41	F	Yes	P	A	R2	20	No			115	Good
10	43	F	No	Re	A	R2	20	No			114	Good
11	50	F	Yes	P	A	R0	12	No			109	Good
12	64	F	No	P	A	R1	20	No			106	Excellent
13	53	F	Yes	P	O	R2	20	No			105	Excellent
14	28	F	Yes	P	O	R1	26	No			97	Excellent
15	49	M	No	P	O	R0	34	No			91	Excellent
16	22	M	No	Re	A	R2	32	No			89	Excellent
17	37	F	No	P	A	R2	32	No			89	Good
18	27	F	No	Re	A	R2	34	Yes	63	Refused	87	Poor
19	22	M	No	Re	O	R2	34	Yes	62	Yes	84	Good
20	31	F	No	P	O	R2	34	No			83	Excellent
21	45	M	No	P	A	R0	26	No			82	Excellent
22	34	F	Yes	P	A	R2	34	No			69	Excellent
23	52	M	No	P	A	R0	34	No			52	Excellent

A Case B Age C Gender D History of trauma E Primary (P) vs. recurrent (Re) F Surgery method A: Arthroscopic O: Open G Resection status R0: No residual disease R1: Suspicion of residual disease R2: Gross residual disease H Dose (Gy) I Recurrence J Disease-free interval (m) K Salvage surgery L Follow-up period (m) M Final joint function

### Treatment characteristics

17 patients underwent arthroscopic synovectomy and the other 6 underwent open synovectomy (Table 1). After cytoreductive surgery, 13 patients had obvious gross residual disease and 2 had suspected residual lesions. The remaining 8 patients, with no macroscopic tumor tissue left after surgery, received adjuvant RT. Patients were given postoperative RT if they had (1) large extra-articular and/or infiltrative disease (n = 10), (2) extensive local recurrences (n = 4), or (3) limited access to the affected joint during arthroscopic surgery (n = 9).

The median interval from the surgical procedure to the commencement of RT was 28 (18–48) days. All patients underwent RT using 4-MV or 6-MV photons with linear accelerators through two opposing fields (anterior to posterior and posterior to anterior) at a dose of 2 Gy per fraction once a day. Of the 23 patients, 13 (cases 1–13) were scheduled to receive 20 Gy by one physician and the other 10 were scheduled to receive 32–34 Gy by the other physician. 2 patients in each group (cases 1, 11, 14, and 21) refused to complete their planned radiotherapy. In four patients, a computed tomography-based 3-dimensional treatment planning system was used. Clinical target volume was defined as the tumor bed plus 5 cm margins superoinferiorly and the entire synovial cavity of the affected knee based on preoperative MRI and/or operation records. The planning target volume was defined from the clinical target volume with a margin of 10 mm.

### Follow-up evaluation and statistical analysis

Following RT, patients were routinely followed up by physical examination, with an MRI performed if symptoms became aggravated. Local recurrence was defined as new or increasing synovial lesions based on either MRI or pathological findings during salvage operations. The functional status of the involved joint was assessed as described previously (O'Sullivan et al. 1995, Berger et al. 2007): excellent (no symptoms), good (minor symptoms), fair (moderate symptoms or joint dysfunction that did not interfere with daily activities), and poor (severe symptoms that interfered with daily activities). Complications of RT were recorded using the Common Terminology Criteria for Adverse Events (CTCAE) version 4.0.

The baseline follow-up date was the start of RT and the last follow-up date was the last hospital visit or date of telephone conversation. Local control was calculated from the baseline date to the date of first recurrence or last follow-up. Univariate analyses were performed to examine the effect on local control of age, sex, history of trauma, primary vs. recurrent disease, number of prior surgeries, and radiation dose (≤ 20 Gy vs. > 20 Gy). All p-values were two-sided, and p-values < 0.05 were considered statistically significant. SPSS software version 12.0K was used for all statistical analyses.

## Results

At a median follow-up time of 9 (0.8–12) years, 19 of the 23 patients showed no evidence of recurrence or persistent disease. 4 patients had local recurrences. 2 had received 20 Gy of external-beam RT and the others had received 34 Gy. Of these 4 patients, 3 regained local control after salvage synovectomy, whereas the remaining patient refused further surgery. The time interval between RT and diagnosis of recurrence was longer than 5 years in 3 of the 4 patients. Univariate analysis showed that age (≤ 37 vs. > 37 years, p = 0.6), gender (p = 1), trauma history (p = 1), primary vs. recurrent (p = 0.1), number of prior surgeries (≤ 2 vs. > 2, p = 0.3), and total dose of RT (≤ 20 Gy vs. > 20 Gy, p = 1) were not associated with rate of local control. Patient and treatment characteristics were similar in the low-dose group and the conventional-dose group (≤ 20 Gy vs. > 20 Gy) (Table 2).

**Table 2.** Patient and treatment characteristics by radiation dose group

Variables	≤ 20 Gy (n = 13)	> 20 Gy (n = 10)	p-value
Mean age, years	38	35	0.6
Male : Female	3:10	5:5	0.2
History of trauma (yes : no)	5:8	2:8	0.4
Primary : Recurrent	12:1	7:3	0.3
Synovectomy method (open : arthroscopic)	2:11	4:6	0.3
Resection status (R0 : R1 : R2)**^ a^**	5:1:7	3:1:6	0.9
Recurrence (yes : no)	2:11	2:8	0.6
7-year recurrence-free survival	83%	78%	0.8
Final joint function (excellent : good : poor)	5:8:0	7:2:1	0.1

**^a ^**Resection status: See Table 1 (G)

Of the 23 patients, 6 reported excellent joint function and 16 reported good joint function, whereas the patient who refused salvage synovectomy reported poor joint function. None of these patients, including the 3 who underwent salvage synovectomy after RT, experienced radiation-related early or late complications greater than grade 2. None developed any radiation-induced malignancy.

## Discussion

Local control was achieved in 19 of the 23 patients who underwent cytoreductive surgery followed by RT, with excellent or good joint function in all but 1 patient. Our findings are similar to those in previous reports (O'Sullivan et al. 1995, Blanco et al. 2001, Chin et al. 2002, Berger et al. 2007, Horoschak et al. 2009, Heyd et al. 2010) (Table 3).

**Table 3.** Previous studies of postoperative external beam radiotherapy

Author	No. of sites (knee)	Median dose (Gy)	Mean follow-up, years (range)	Local control **^a^** of knee lesion (%)
O'Sullivan **^b^** et al. 1995	14 (4)	35	5.8 (1.1–21)	100 **^c^**
Blanco **^b^** et al. 2001	22 (22)	26	2.8 (2.2–6.3)	86
Chin **^d^** et al. 2002	5 (5)	35	3.1 (2.7–3.5)	60
Berger et al. 2007	7 (5)	40	2.4 (0.3–9.3)	100
Horoschak et al. 2009	16 (12)	34	3.8 (0.7–15)	75 **^f^**
Heyd **^e^** et al. 2010	41 (25)	36	NA (0.5–10≠)	95 **^f^**
This study	23 (23)	20	8.3 (0.8–12)	83

NA: not available.

**^a^** crude rate.

**^b^** mainly using cobalt-60 irradiation.

**^c^** including 1 patient with stable disease.

**^d^** including postoperative external beam radiotherapy group only.

**^e^** One patient received radiotherapy with an orthovoltage X-ray machine and 2 patients with a cobalt-60 machine.

**^f^** Local control rate for the entire cohort.

There are several distinguishing aspects of our analysis. First, the follow-up time (median 9 years) was far longer than in most other studies. Long follow-up is required to detect local recurrence. For example, the average time to local recurrence in 4 patients (Horoschak et al. 2009) was 3 years, and local recurrences have been reported as long as 17 years after primary treatment (Panagiotopoulos et al. 1993). In our study, the 4 patients with recurrent disease had a median time to failure of 5 years, with 3 having disease-free intervals of longer than 5 years. Secondly, the median total dose of postoperative RT in our patients was 20 Gy, which was much lower than in other studies (O'Sullivan et al. 1995, Blanco et al. 2001, Chin et al. 2002, Lee et al. 2005, Berger et al. 2007, Horoschak et al. 2009, Heyd et al. 2010). The doses as low as 20 Gy in 10 fractions came from the general prescribed doses for benign diseases such as thyroid ophthalmopathy (Petersen et al. 1990), orbital pseudotumor (Lanciano et al. 1990), and heterotopic ossification (MacLennan et al. 1984). Treatment of PVNS with total doses of < 30 Gy has been associated with recurrence rates of 4–14% after median 3.5 years of follow-up (Ustinova et al. 1986, Kotwal et al. 2000, Blanco et al. 2001). Although other studies have used doses of 30–50 Gy (O'Sullivan et al. 1995, Berger et al. 2007, Heyd et al. 2010), there have been no studies showing dose-response relationships for postoperative RT in patients with PVNS. A recent study examining the correlation between total dose and local control in 41 patients from 14 departments failed to show a significant dose-effect relationship (Heyd et al. 2010). Of the 4 patients in our study who experienced recurrences, 2 had received 20 Gy RT whereas the other 2 had received 34 Gy, which indicates the same local control rate for the two treatment regimens. Thirdly, only patients with PNVS of the knee joint were included in our analysis. Despite the inconclusive relationship between location of PVNS and local control rate, local recurrence of PVNS of the knee has been reported more frequently than recurrences at other joints (Schwartz et al. 1989).

We found similar local control rates in patients with primary PVNS and those with recurrent PVNS. Likewise, the number of previous surgeries had no effect on local control and functional outcomes. These findings are in accordance with those from a study of 24 patients with primary PVNS and 17 patients with recurrent PVNS (Heyd et al. 2010). Use of RT for benign disease such as PVNS may introduce concerns about the risks of functional impairment and radiation-induced malignancy. Of our patients, all but 1 showed excellent or good joint function. Similarly to other small-sized series, we did not observe any severe early or late radiation-related complications or secondary malignancies. To our knowledge, there have been no reports to date of radiation-induced malignancy after RT for PVNS.

The limitations of our study were its retrospective design, which may have introduced selection bias, and the small number of patients. Due to the low incidence of PVNS, it is almost impossible to perform large-scale prospective studies. To our knowledge, our study is the largest one so far of patients at a single center who received RT with modern technique for diffuse PVNS of the knee, and showing good local control.
